# Vertical-flow tearable paper-tape rolls for scalable multiplexed point-of-care nucleic acid testing

**DOI:** 10.1038/s41378-026-01172-w

**Published:** 2026-04-07

**Authors:** Shaorui Shi, Zhiying Wang, Yongchao Yao, Yue Gao, Jianchao Tang, Chuyan Zhang, Hao Bai, Zhao Du, Jie Hu, Yue Su, Guannan Chen, Xinxia Cai, Binwu Ying, Lingqian Chang, Wenchuang Hu, Yang Wang

**Affiliations:** 1 Department of Laboratory Medicine, West China Hospital, Sichuan University, Chengdu, China; 2 Department of Laboratory Medicine, The Second People,s Hospital of Yibin, West China Hospital, Yibin Hospital Sichuan University, Yibin, China; 3 Precision Medicine Translational Research Center, West China Hospital, Sichuan University, Chengdu, China; 4 Beijing Advanced Innovation Center for Biomedical Engineering, School of Biological Science and Medical Engineering, Beihang University, Beijing, China; 5 Med+X Center for Manufacturing, West China Hospital, Sichuan University, Chengdu, China; 6 Institute of Solid Mechanics, Beihang University, Beijing, China; 7 School of Mechanical Engineering, Sichuan University, Chengdu, China; 8 State Key Laboratory of Transducer Technology Aerospace Information Research Institute, Chinese Academy of Sciences, Beijing, China; 9 University of Chinese Academy of Sciences, Beijing, China; 10 School of Engineering Medicine, Beihang University, Beijing, China

**Keywords:** Nanoscale devices, Chemistry

## Abstract

Paper substrates, with their inherent capillary-driven forces, eliminate the need for external power sources typically required by microfluidic biochips. However, conventional lateral flow (LF) systems face limitations, including non-specific adsorption, uneven molecular distribution, and restricted flexibility for multiplexed detection. In this work, we developed a novel vertical-flow paper-tape (VFPT) integrated device. By engineering a gradient pore-size paper substrate and replacing pre-patterned channels with gravity-driven flow, this innovation achieves size-selective molecular transport while doubling the effective transport distance. Its roll-to-roll fabrication process enables scalable, low-cost production with customizable detection capabilities. As a proof of concept, the VFPT device demonstrated detection limits of 200 copies/mL for HIV and HBV and 600 copies/mL for HCV, comparable to PCR and superior to traditional paper-based systems. Validation with 203 blinded clinical plasma samples revealed sensitivity and specificity exceeding 90.9%. This innovative platform offers a user-friendly, accurate, and cost-effective solution for clinical diagnostics and resource-limited settings.

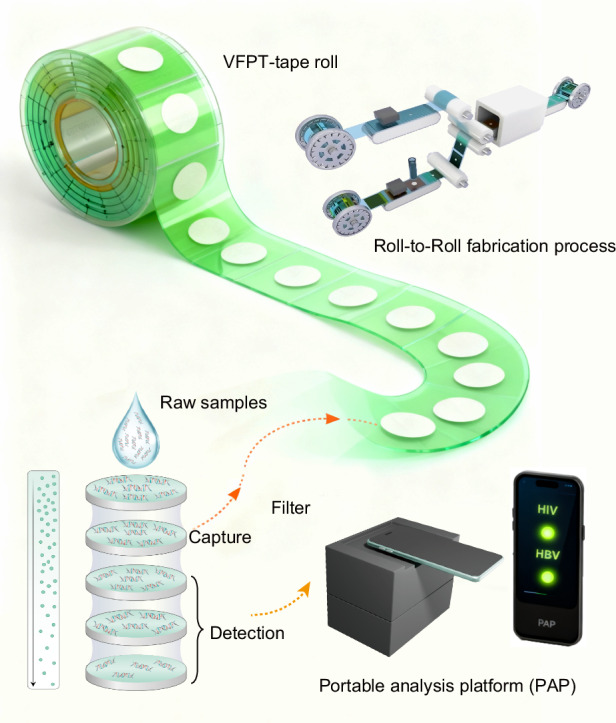

## Introduction

Paper substrates possess inherent capillary-driven forces, eliminating the heavy reliance on external power sources typically required by microfluidic biochips^1–3^. This distinctive feature makes them uniquely suited for enhancing low-cost, portable point-of-care diagnostic (POCT) devices, and offers remarkable advantages over traditional substrates like glass, silicon, or polydimethylsiloxane (PDMS)^4,5^. Over the past decades, paper-based microfluidic technologies have advanced rapidly, with their global market value exceeding 6.5 billion USD^6,7^.

In paper-based microfluidic chips, capillary forces typically drive samples to flow laterally through pre-patterned channels, enabling functions such as mixing, separation, amplification, and detection^8,9^. A classic example is the lateral flow (LF) chromatography strip, which facilitates qualitative detection of analytes such as biomarkers, pathogens, or contaminants^10^. Yuguo Tang reported an amplification-free nucleic acid immunoassay implemented on a lateral flow strip, which enables the detection of SARS-CoV-2 RNA in less than one hour. This assay eliminates the need for external pumps, highlighting its potential for application in resource-limited settings^11^. However, the lateral flow paradigm encounters intrinsic technical limitations in molecular transportation, which compromise detection accuracy and sensitivity: (1) capillary diffusion increases non-specific adsorption of trace molecules onto the cellulose within channels, reducing the sample yield in the detection area. Without compensation, this ultimately impacts detection accuracy and sensitivity^5,12^; (2) lateral flow often induces the “coffee ring effect,” where molecules aggregate along droplet edges, forming ring-like precipitations that result in uneven molecular distribution, diminishing detection accuracy, reproducibility, and quantitative potential^13,14^; (3) lateral flow designs impose fixed capacities determined by pre-patterned channels, limiting flexibility in multiplexed testing^15,16^. By contrast, there exists a category of paper-based systems driven by gravity-induced flow. For instance, Jonathan M. Cooper developed a vertical flow paper device for malaria DNA detection^8^. The system integrated with a LAMP detection platform, greatly enhanced sample processing efficiency. However, these vertical flow systems lack a gradient pore-size design for size-selective molecular transport, and their fabrication methods (e.g., hand cutting, small-batch stamping) limit scalability and multiplexing capabilities.

Therefore, in this work, we introduce a pure vertical flow (VF)-based paper-fluidic system, which uses gravity to drive the liquid flow through the gradient pore-size paper based material, to achieve the selective transport of molecules, significantly reducing non-specific adsorption and enhancing molecular transport efficiency. Based on this principle, a full-process POCT platform integrating sample processing, nucleic acid extraction, isothermal amplification and intelligent analysis was developed. This system was applied to clinical DNA diagnostics, achieving detection limits of 200 copies/mL for HIV and HBV, and 600 copies/mL for HCV—results comparable to commercial PCR methods and far superior to traditional LF paper-based devices. Mathematical modeling and experimental data corroborated the uniform molecular delivery of the system, as evidenced by consistent gray values and fluorescence intensities. PCR analysis of nucleic acids collected from each layer revealed significantly lower cycle threshold (Ct) values for VF systems compared to LF designs. The VF system leverages gravity-driven pressure gradients to achieve a twofold increase in transportation distance for various molecules (e.g., nucleic acids, proteins, fluorescein sodium). Its origami-inspired design further enables highly scalable multiplexing capabilities.

Additionally, we developed a roll-to-roll production method that integrates paper and tape as substrate materials^9^, enabling high-throughput fabrication of vertical-flow paper-tape (VFPT) devices. This approach reduces production costs and enhances scalability for multiplexed detection. The VFPT device is user-friendly by combining a custom portable detector for isothermal expansion and a smartphone app for real-time quantitative analysis. Validation of 203 blinded clinical plasma samples demonstrated a sensitivity and specificity exceeding 90.9%, underscoring the VFPT device’s potential as a practical, economical solution in clinical and resource-limited settings.

## Results

### Structure and working principle of the VFPT

**Fig. 1:**
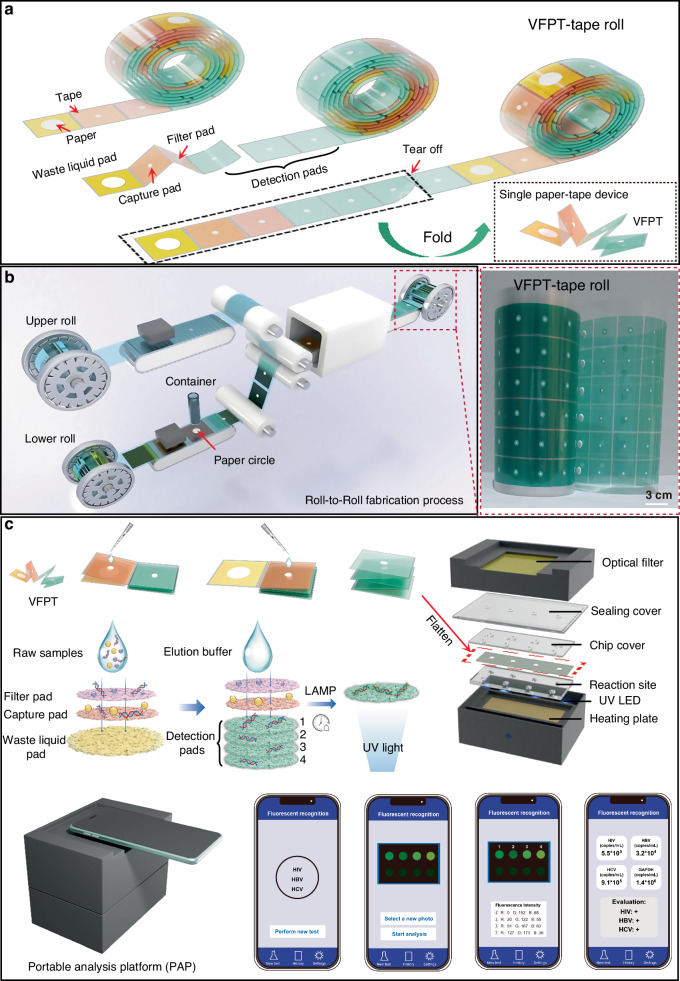
Structure, fabrication, and detection process of the VFPT-based analytical platform. **a** VFPT-tape roll: The VFPT is prepared as a roll, similar to tape. The colored areas are made of tape, and the white circular areas are cellulose filter paper. A single VFPT, with multiple functional pads (3 cm per side), is torn off and folded for use. **b** Roll-to-Roll Fabrication: The VFPT is mass-fabricated using a roll-to-roll process, with the fabricated VFPT-roll shown in the image. **c** VFPT-Based Platform Workflow: The VFPT is used for sample preparation and nucleic acid extraction through four steps via vertical flow. The folded multiplexed detection pad is then flattened and placed on the homemade portable analysis platform (PAP) for isothermal amplification. Results are quantitatively read and analyzed through a smartphone app

The VFPT is designed as a tape roll, with its structure detailed in Fig. 1a. It is composed of two low-cost materials: paper (represented by the white circles) and tape (denoted by the colored areas). The tape serves as both a hydrophobic and structural support material for the device. Each VFPT unit consists of four programmable functional components, each measuring 3 cm on each side. These components include a waste liquid pad (yellow), a capture pad (orange), a filter pad (pink), and multiple detection pads (green). The device can be easily separated from the VFPT roll to create individual units of adjustable length, based on the specific requirements of different point-of-care testing (POCT) scenarios. Specifically, each torn-off unit is for single-sample testing and discarded after use, which fundamentally prevents cross-contamination between different samples. The length of the VFPT determines the number of detection pads, enabling scalable multiplexed detection. When folded, the VFPT facilitates nucleic acid-based testing, covering sample preparation, amplification, and detection.

The VFPT is manufactured using a roll-to-roll method, allowing for high-throughput production (Fig. 1b, Movie S1). This process involves two rolls of tape - upper and lower - that seal a circular paper layer at the center. The combination of low-cost paper and tape, together with the roll-to-roll mass production technique, reduces the cost of each device to just $ 0.10, providing an affordable and scalable solution for paper-based devices with strong commercialization potential.

The VFPT enables precise, quantitative nucleic acid testing on-site, using a custom portable analysis platform (PAP) and smartphone, in four simple steps (Fig. 1c). Using a paper-based material with a gradient aperture, the full-process POCT platform, which integrates sample processing, nucleic acid extraction, isothermal amplification and intelligent analysis, was carried out. First, raw samples are added to the VFPT, which is then folded to activate filtration through the pink filter pad. Purified nucleic acids are captured on the orange capture pad, while waste liquids flow downstream to the waste liquid pad at the bottom. To transfer the nucleic acids to the detection sites, the folded capture pad is flipped over, positioning it above the multiple detection pads. The addition of an elution buffer allows the nucleic acids to be transferred directly to the detection pads with minimal loss. The number of detection pads can be varied to support scalable multiplexed analysis.

The folded detection pads are then flattened and transferred to the portable analysis platform (PAP). This device contains pre-deposited species-specific LAMP reagents - primers, enzymes, fluorescent dyes, and reaction mixtures. With dimensions of 150 × 105 × 121 mm and a weight of 250 g, it is portable and easy to transport (Fig. S1A). The amplification sites are sealed with a chip cover and a sealing cover to form completely sealed chambers, separating the amplification chambers from the external environment to prevent aerosol contamination (Fig. S1B). Powered by a 3.7 V lithium battery, the portable device maintains a constant amplification temperature of 65 °C, which is required for LAMP, through an embedded heating plate. This eliminates the need for an external power source, making the device ideal for resource-limited settings (Fig. S1C). The LAMP reaction is triggered by adding target nucleic acids extracted by the VFPT. After amplification, fluorescent signals are generated by excitation from embedded UV-LEDs, which are directly read by a smartphone app. This ensures the chamber remains sealed throughout the detection process, avoiding post-amplification nucleic acid exposure and potential contamination of subsequent tests. Quantitative results are then calculated and displayed on the screen (Movie S2). Together, the low-cost VFPT, portable analysis platform (PAP) and smartphone provide an effective solution for point-of-care nucleic acid testing.

### Characterization of the vertical flow approach

**Fig. 2:**
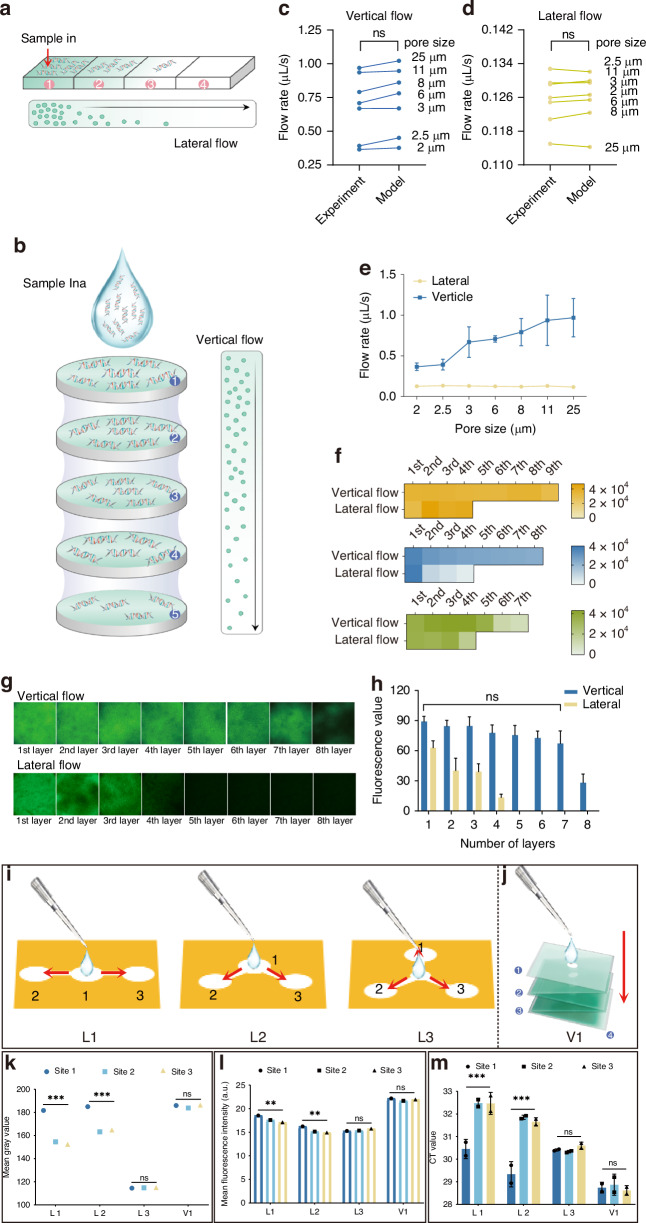
Experimental results: vertical flow vs. conventional lateral flow. **a** VF Schematic: Liquid samples are added from the top and flow downward. **b** LF Schematic: Liquid samples are added from one side and flow across to the other side of the device. **c** Vertical Flow Velocity: Comparison of experimental flow velocity with the theoretical model. **d** Horizontal Flow Velocity: Comparison of experimental flow velocity with the theoretical model. **e** Vertical vs. Horizontal Flow Velocity: Direct comparison of velocities in both flow directions. **f** Transport Distance Comparison: fluorescein sodium (orange), fluorescently labeled HIV plasmids (blue, 1500 bp), and Cy5-conjugated BSA (green, 66 kDa) were used to compare transport distances in VF and LF. **g** Fluorescent Images: VF and LF transport of fluorescently labeled HIV plasmids across different layers. **h** Fluorescence values fluorescently labeled HIV plasmids of different layers in VF and LF transport between different layers. **i** Lateral Flow Configurations: Diagrams of three lateral flow setups (L1-L3). **j** Vertical Flow Setup: Illustration of the vertical flow setup. **k** Comparison of Four Flow Methods: Comparison of four flow configurations (L1-L3, V1) for mean gray values at each site. **l** Fluorescence Intensity: Comparison of mean fluorescence intensity at each site. **m** PCR Analysis: Real-time PCR results based on nucleic acids collected from each site

The VFPT employs a pure vertical flow (VF) mechanism (Fig. 2a), as opposed to the lateral flow (LF) used in conventional paper-based devices (Fig. 2b). During the VF test, the pressure gradient of the fluid is mainly caused by the remaining volume of fluid $V\left(t\right)$ on the upper side of the cellulose filter papers according to the Darcy's law flow^16^. By denoting the initial fluid volume in the test as ${V}_{0}$, $V\left(t\right)$ is determined as

$$V\left(t\right)={V}_{0}\exp \left(-\frac{\rho {gk}}{\mu h}t\right)$$

where ${k}$ is a constant permeability as Darcy flow, $\mu$ denotes the fluid viscosity, $h$ denotes the thickness of the filter pad, $\rho$ denotes the density of the liquid sample, and $g$ denotes the local gravitational acceleration. Once the volume of the remaining liquid is lower than a specific threshold ${V}_{{end}}=\phi {V}_{0}$, the test is stopped and the average volumetric flow rate $\bar{{v}_{{vf}}}=\bar{q}S$ of the vertical flow is calculated as

$$\bar{{v}_{{lf}}}=\frac{\mu {S}_{0}{V}_{0}}{\rho g\gamma k{S}_{1}^{2}\left[{V}_{0}\mathrm{ln}\left(\frac{{V}_{0}}{{V}_{0}-\gamma {S}_{1}{L}_{0}}\right)-\gamma {S}_{1}{L}_{0}\right]}$$

In the LF case, the fluid sample diffuses in the $x$-axis, and the horizontal volumetric fluid flux $q\left(x,t\right)$ follows Darcy’s law and the principle of conservation of mass^17,18^. The fluid-saturated length is calculated as

$$L\left(t\right)=\frac{{V}_{0}}{\gamma {S}_{1}}\left\{1+{W}_{0}\left[-\exp \left(-1-\frac{\rho g\gamma k{S}_{1}^{2}}{\mu {S}_{0}{V}_{0}}t\right)\right]\right\}$$

Where $\gamma$ denotes the volume of fluid required to infiltrate a unit length of cellulose paper, ${S}_{1}$ denotes the vertical cross-section of the cellulose paper, ${W}_{0}(z)$ denotes the Lambert W function, and ${S}_{0}$ denotes the initial area occupied by the fluid drip. And the average volumetric flow rate $\bar{{v}_{{lf}}}$ is calculated.

With different types and layers of the cellulose filter papers used, the permeabilities of the cellulose papers are calibrated^19^. The model’s predictions matched experimental flow velocities of VF and LF (Fig. 2c, d), confirming that vertical flow outperforms horizontal flow in flow rates and finally processing efficiency (Fig. 2e). A key advantage of VF is that gravity-driven fluid flow dominates over diffusion, which ensures particles are transported to detection pads with the fluid. As derived from Darcy’s law, the flow velocity of nucleic acid molecules in VF exceeds 0.4 μL/s, while that in LF is only ~0.1 μL/s, meaning VF flow rate is at least 4-fold higher than LF. This rapid downward flow drives molecules to move with the fluid, avoiding significant lateral diffusion. Thus, the VF mechanism enables longer mass transport distances within the filter paper compared to LF for the same volume of liquid within the same period (Fig. 2f). As shown, the VF transport distance is nearly twice as long as LF for various molecules, including fluorescently-labeled nucleic acids (HIV plasmids, 1500 kb) (blue), proteins (Cy5-conjugated bovine serum albumin, Cy5-BSA, 66 kDa) (green), and fluorescein sodium (orange), using filter paper with an approximate pore size of 8 μm (Fig. 2f). Additionally, the transport distance is positively correlated with molecular weight, as smaller molecules (e.g., fluorescein sodium) travel further (to the 9th layer), while larger molecules (e.g., HIV plasmids) travel less far (to the 8th layer), and Cy5-BSA reaches the 7th layer. This demonstrates that smaller molecules travel longer distances. Further validation with filter papers of varying pore sizes (ranging from 1 μm to 25 μm) and the three types of molecules yields similar results, confirming that VF consistently outperforms LF in transport efficiency (Fig. S2).

Moreover, the particles in the fluid samples in the VF are considered transported to the detection pads with the fluid rather than diffusing to each pad. Thus the nucleic acid of the samples is considered evenly transported. This assumption was verified directly by the fluorescence images taken for different layers of the filter pads given in Fig. 2g. No significant difference in fluorescence intensity is observed, indicating consistent transport. This contributes to a more uniform detection compared to LF (Fig. 2h). In contrast, LF exhibits a strong intensity gradient, with the highest fluorescence in the first layer and a gradual decrease in subsequent layers.

Next, we compared the actual performance of VF (V1) with three commonly used LFs (L1-L3), each with three detection sites (Fig. 2i, j). Twenty microliters of red ink and fluorescein sodium were added simultaneously to the four devices. Both the magnitude and uniformity of mean gray values and fluorescence intensity were significantly better for VF compared to the LFs (Fig. 2k, l). We then used liquid samples containing the same concentration of nucleic acids. Nucleic acids collected from each detection site on the devices were analyzed by polymerase chain reaction (PCR). The cycle threshold (Ct) values for VF were significantly lower than those for the three LFs (Fig. 2m), demonstrating the superior transport efficiency of VF.

### Characterization of the VFPT

**Fig. 3:**
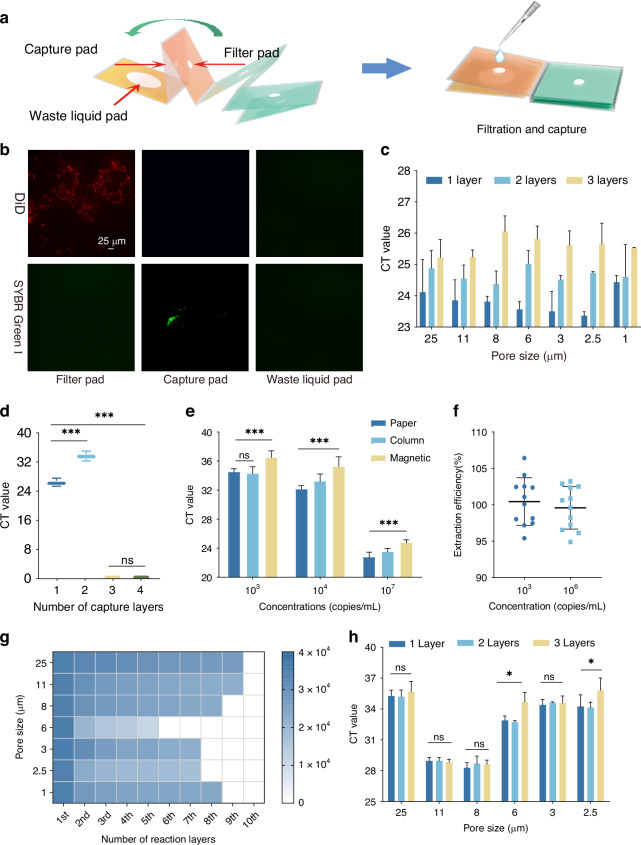
Characterization of the prepared VFPT. **a** Schematic of Filtration and Capture: The VFPT’s filtration and capture process is illustrated. **b** Fluorescence Detection: Fluorescence images of the filter pad, capture pad, and waste liquid pad show the filtration and capture functions (scale bar: 25 μm). **c** Filter Pad Optimization: Results of optimizing the number of layers and porosity of the filter pad. **d** Capture Pad Optimization: Results of optimizing the number of layers of the capture pad. **e** Extraction Efficiency Comparison: Comparison of extraction efficiency between VFPT-based, column-based, and magnetic bead-based sample preparation methods. **f** Extraction Efficiency Validation: Validation of extraction efficiency using 10^3^ and 10^6^ copies/mL samples with the VFPT. **g** Vertical Flow Transmission Distance: Distance traveled by liquid samples in the vertical flow setup. **h** Detection Pad Porosity Optimization: Results of optimizing the porosity of the detection pads

The VFPT enables raw sample filtration and nucleic acid extraction via a simple origami structure (Fig. 3a). To validate this dual function, we used whole blood samples stained with DiD (a far-infrared fluorescent dye for cell membranes) and spiked with SYBR Green I-labeled nucleic acids. After lysis, the liquid flowed vertically from the filter pad through the capture pad to the waste pad. The filter pad effectively isolated DiD-stained cell membranes, preventing debris from entering downstream components and avoiding non-specific adsorption of nucleic acids to impurities. Meanwhile, fluorescently labeled nucleic acids were concentrated exclusively in the silica membrane capture pad, with no detectable fluorescence in the filter or waste pads (Fig. 3b). This outcome confirms the synergistic function of the two pads: the filter pad retains cell debris to avoid non-specific adsorption of nucleic acids to impurities, while the silica membrane capture pad specifically binds nucleic acids via hydrophobic and electrostatic interactions, minimizing loss of target molecules. To optimize performance, we tested various filter pad configurations (material, layers, pore size). Single-layer cellulose filter papers (1–25 μm pore size) resulted in significantly lower Ct values than multi-layer pads (Fig. 3c), therefore, we selected a 6 μm pore filter for subsequent experiments. For the capture pad, we evaluated the impact of layer count on extraction efficiency and determined that two layers was optimal (Fig. 3d).

We compared VFPT’s performance to commercial column-based (BW-VR6511, Beiwo) and magnetic bead-based (R6664-01, Magen) methods using 10^3^, 10^4^, and 10^7^ copies/mL samples. For low concentrations (10^3^ copies/mL), VFPT performed similarly to column-based methods and slightly better than magnetic beads. At higher concentrations (10^4^–10^7^ copies/mL), VFPT outperformed both methods, showing lower Ct values (Fig. 3e). Extraction efficiency exceeded 95% across concentrations (Fig. 3f). VFPT also handled varying liquid volumes, with Ct values decreasing from 33.61 to 30.06 as the volume increased from 150 μL to 900 μL (Fig. S3). Unlike commercial methods, VFPT requires no external instruments or power, making it a low-cost, efficient solution for point-of-care nucleic acid testing.

For detection, extracted nucleic acids are eluted onto detection pads for amplification. The number of detection pad layers enables scalable multiplexing, eliminating the need for lateral fluidic channels. We investigated the volume of liquid required for adequate wetting across different layer counts, which increased proportionally with the number of layers (R² = 0.997) (Fig. S4A). For each layer configuration, a precise liquid volume minimizes loss due to non-specific adsorption and waste, ensuring high nucleic acid yield. VF allowed nucleic acids to transfer across detection sites on multiple layers. For fixed sample volumes (20 μL), transportation distance exceeded 7 layers for most filter papers, except for the 6 μm pore size, which had a higher Cobb value (Fig. 3g, S5). This indicates that, with a limited sample volume, VFPT enables higher detection throughput.

Various filter papers were tested as detection pads, and the 8 μm pore size performed best, yielding the lowest Ct values and minimal variation between layers (*P* > 0.05) (Fig. 3h). The first seven layers showed stable Ct values for both low (10^3^ copies/mL) and high (10^7^ copies/mL) concentrations (Fig. S4B). This filter was selected for further use.

### Characterization of isothermal amplification

For sensitive and specific detection of HIV, HBV, and HCV, LAMP primers targeting their specific sequences were designed and optimized (Table S1). Sequencing analysis showed >95% consistency with target sequences, and electrophoresis confirmed the correctness of LAMP primers via clear target amplification bands (Fig. S6). Cross-reactivity detection with 10 interfering viruses (e.g., HBV, HCV, HPV, Influenza) was performed via real-time LAMP curves, which showed no amplification with the HIV, HBV, or HCV primers (Fig. S7A–C). The presence of these viruses had minimal impact on amplification, with no significant change in Ct values (*P* > 0.05) (Fig. S7D–F).

To address the quantitative capability of LAMP, we employed an endpoint detection strategy using EvaGreen, a double-stranded DNA (dsDNA)-binding dye that interacts with dsDNA via a “reverse intercalation” mode^11,20,21^. Under a reaction time of 60 minutes, samples with higher initial DNA concentrations accumulate more dsDNA, while low-concentration samples show relatively less product accumulation. Sensitivity testing showed limits of detection (LOD) of 200 copies/mL for HIV and HBV, and 600 copies/mL for HCV, with a linear range from 10^2^ to 10^8^ copies/mL (R² > 0.99), comparable to real-time PCR (Fig. S8A–D).

**Fig. 4:**
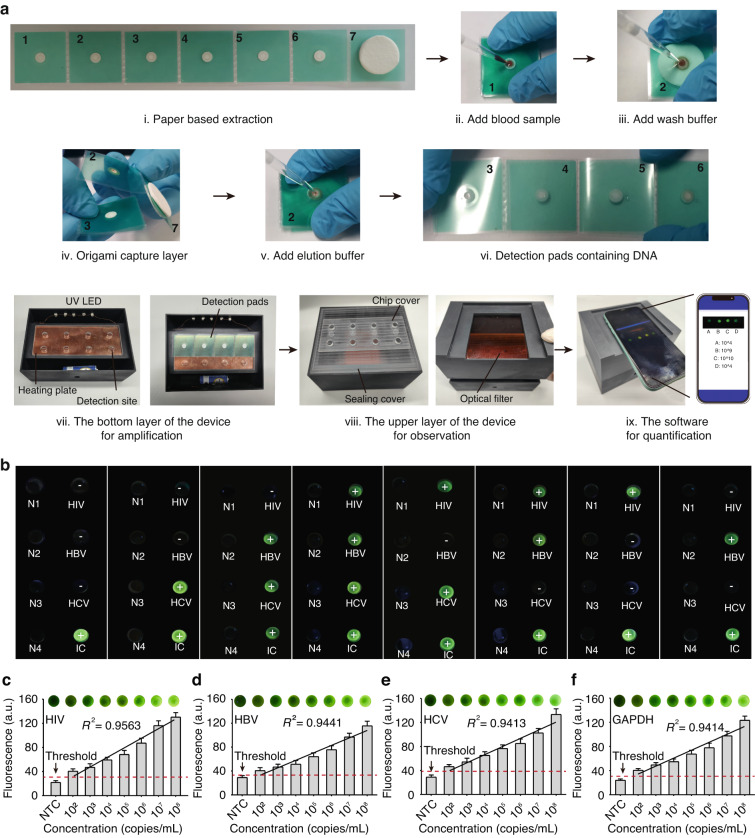
Detection performance of the VFPT. **a** Operation Steps: Illustration of sample preparation, multiplexed amplification, and detection on the VFPT platform. **b** Amplification Results: Pictures showing simultaneous amplification for HIV, HBV, and HCV. Positive control: GAPDH; Negative control: ddH₂O. **c**–**f** Sensitivity and Detection Range: Quantization results of fluorescence value for HIV, HBV, HCV, and GAPDH detection of VFPT

A VFPT-based analysis platform, including a portable amplification device and smartphone, was developed for point-of-care testing (POCT). The battery-powered platform maintains a constant temperature of 65 °C for LAMP and uses UV LEDs to capture fluorescence, with quantitative results displayed via a custom app. To achieve reliable quantification, the smartphone app detects fluorescence signals generated by the LAMP reaction; based on the linear relationship between fluorescence intensity and DNA/RNA concentration, quantitative information is derived. To simultaneous detection of HIV, HBV, and HCV, a VFPT with four detection pads (each corresponding to one of the targets and a positive control) was processed for nucleic acids extraction (Fig. 4a). The platform demonstrated a linear range of 10^2^ to 10^8^ copies/mL and a LOD of 10^2^ copies/mL for all three viruses (Fig. 4c–e). The same performance was achieved with plasma samples, confirming VFPT’s efficient extraction.

**Fig. 5:**
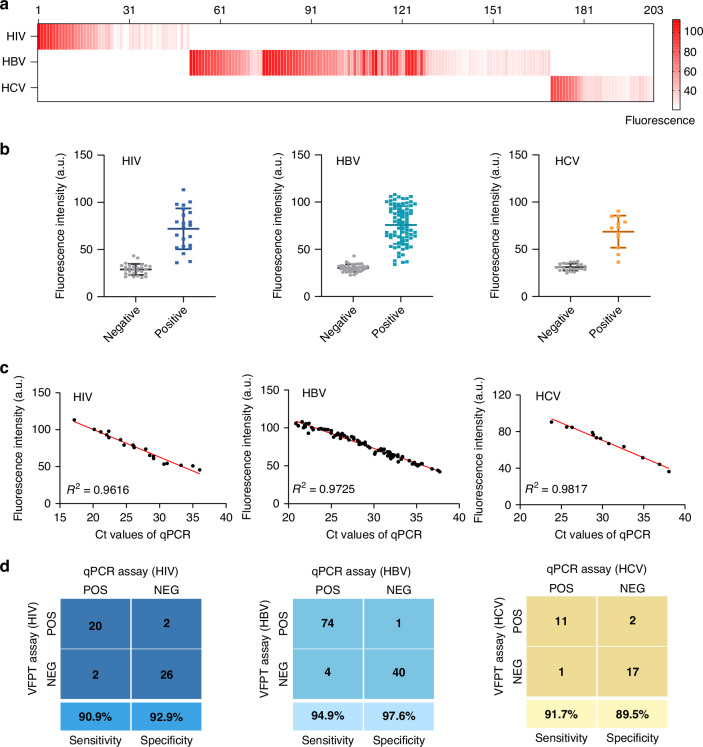
Detection of clinical plasma samples using VFPT for the diagnosis of different diseases. **a** Fluorescence values for each of the 203 samples. **b** Comparison of fluorescence values between positive and negative samples. **c** The fluorescence value from VFPT exhibits a strong linear correlation with the Ct value from qPCR. **d** Sensitivity and specificity of VFPT compared to qPCR

To evaluate the clinical performance of the VFPT platform, we tested it with 203 plasma samples from West China Hospital, Sichuan University, and Yibin Hospital. The samples included 50 for HIV, 119 for HBV, and 31 for HCV detection. The fluorescence values for each sample are shown in Fig. 5a. Notably, the fluorescence intensity of the positive samples was significantly higher than that of the negative ones (Fig. 5b). Furthermore, when compared to the Ct values from qPCR, samples with higher fluorescence intensities exhibited lower Ct values, showing a strong linear correlation and confirming the consistency between the two methods (Fig. 5c).

In addition, qPCR was used as the gold standard to verify VFPT’s accuracy. The platform correctly identified 20 out of 22 HIV-positive cases, 74 out of 78 HBV-positive cases, and 11 out of 12 HCV-positive cases. The sensitivity of VFPT for HIV, HBV, and HCV was 90.9%, 94.9%, and 91.7%, respectively, while the specificity was 92.9%, 97.6%, and 90.9%, as shown in Fig. 5d. The VFPT-based platform shows strong potential for widespread use in POCT for nucleic acid-based disease diagnostics.

## Discussion

In this work, we present a novel VFPT-based platform for on-site nucleic acid detection, integrating a portable device powered by a battery and coupled with a smartphone application. This system represents a significant departure from conventional workflows, eliminating the need for centralized laboratories, expensive instruments, and external power sources. It enables complete nucleic acid analysis, encompassing sample filtration, lysis, nucleic acid extraction, isothermal amplification, and quantitative detection. This streamlined process opens up new possibilities for POCT applications, particularly in emergency or resource-constrained settings, where access to traditional laboratory infrastructure may be limited.

A critical challenge for paper-based devices intended for point-of-care diagnostics is ensuring robust detection performance, including user-friendly functionality, sensitivity, and the ability to detect multiple targets simultaneously^22–24^. The VFPT platform addresses these challenges by leveraging a unique VF approach. This design allows for higher multiplexing capabilities, as detection throughput can be easily adjusted by varying the number of detection pad layers, in contrast to traditional lateral flow assays that rely on pre-designed fluidic channels with fixed throughput. Moreover, the VFPT minimizes non-specific adsorption and nucleic acid loss, resulting in higher yield and improved detection sensitivity. When applied to the detection of HIV, HBV, and HCV, the platform achieved a limit of detection (LOD) as low as 10^2^ copies/mL, with a wide dynamic range spanning from 10^2^ to 10^8^ copies/mL for each virus. These findings were validated using 203 clinical samples in a blinded study, demonstrating the platform’s high sensitivity and specificity. Notably, the VFPT system is not limited to viruses, by modifying the lysis buffer (e.g., adding lysozyme to break down bacterial cell walls), it can also detect bacteria. Additionally, compared to costly real-time qPCR instruments ($15,000–$30,000) requiring stable AC power, the VFPT uses a 3.7 V lithium battery-powered portable analysis platform (PAP) coupled with a smartphone, better suiting resource-limited scenarios.

The cost-effectiveness of the VFPT platform, combined with its scalability, makes it well-suited for mass production and practical application. Unlike conventional fabrication methods such as hand cutting or small-batch stamping, which are labor-intensive and unsuitable for large-scale production, our approach utilizes low-cost materials—paper and tape—fabricated through a roll-to-roll process^25,26^. This scalable method provides an alternative to more expensive techniques such as laser cutting or photolithography, which require costly equipment and materials^27–30^. Although wax printing was once considered a promising low-cost alternative, its discontinuation has highlighted the need for innovative approaches, such as our VFPT system, that balance ease of fabrication with performance. Building on this foundation, modifications to the paper-based chip are expected to further enhance the sensitivity of point-of-care testing (POCT)^31,32^. For example, based on the nucleic acid-binding mechanism of the current capture layer, weak zwitterionic polymers can be modified onto the silica surface of the capture layer, which not only improves the extraction of nucleic acids, but also reduces the non-specific adsorption of proteins, cell debris, or residual lysate components^33^.

In conclusion, the VFPT-based platform combines portability, scalability, and cost-efficiency with excellent detection performance, making it a promising candidate for on-site nucleic acid detection. Its ability to operate independently of centralized laboratories positions it as an ideal tool for early disease diagnosis, particularly in resource-limited settings or in situations requiring rapid response.

## Methods

### Materials and reagents

The cellulose filter papers with different pore sizes (25 μm, 11 μm, 8 μm, 6 μm, 3 μm, 2.5 μm) required for VFPT were purchased from Whatman (Cytiva, UK). The silicon film for the capture pad was obtained from Biocomma (Shenzhen, China). The lysate and washing buffer used for nucleic acid extraction in the VFPT were supplied by Beiwo (Hangzhou, China).

The HIV standards were obtained from Beijing Conchstein Biotechnology Co., Ltd., the HBV standards were sourced from Bio BDS (Guangzhou, China), and the HCV standards were provided by Sansure Biotech (Changsha, China). The primers and plasmids for HIV, HBV, HCV, and GAPDH were synthesized by Sangon Biotech (Shanghai, China). The primers are listed in Tables S1 and S2. DNA solutions for Human papillomavirus, Influenza A virus, Influenza B virus, Ureaplasma urealyticum, Chlamydia trachomatis, Gonococcus, Mycobacterium tuberculosis, and Group B Streptococcus were provided by the Laboratory of West China Hospital, Sichuan University, Yibin Hospital.

The components of the LAMP system include MgSO₄, KCl, NaOH, and DEPC-ddH₂O, all purchased from Sangon Biotech (Shanghai, China). dNTP-Mix and RNA inhibitors were sourced from Yeasen Biotech (Shanghai, China), while Bst DNA/RNA polymerase was provided by Nuhigh Biotech (Suzhou, China). Reverse transcriptase (1500 U/μL) came from Fapon Biotech (Dongguan, China), and EvaGreen fluorescent dye (20×) was purchased from Jiqi Biotech (Shanghai, China).

### Design and fabrication of the VFPT

VFPT consists of two low-cost materials: cellulose filter papers and PET tapes. During the production process, circular cellulose filter papers are sealed in the center of the upper and lower rolls of tape. Using a hot-pressing device, the VFPT with various functional parts can be sequentially formed. Each VFPT comprises four programmable functional parts, each with a side length of 3 cm, including a waste pad (yellow), a capture pad (orange), a filter pad (pink), and a variable number of detection pads (green).

The different functional parts incorporate cellulose filter papers made from various materials: 100% cotton fiber for the waste liquid pad (diameter 22 mm), silicon films for the capture pad, cellulose papers with an aperture of 6 μm for the filter pad, and cellulose papers with an aperture of 8 μm for the detection pads. Rapid nucleic acid extraction is facilitated by the easy folding of the functional pads.

### Design and fabrication of portable analysis platform

The portable analysis platform (PAP) was designed using AutoCAD 2020 and SolidWorks 2017 as an accessory to the VFPT platform. Measuring 150 mm in length, 105 mm in width, and 121 mm in height, the device was manufactured using 3D printing.

The device is divided into two layers: the lower layer serves as the LAMP amplification area for nucleic acids extracted from the VFPT, while the upper layer is the observation area for collecting fluorescence from the amplification products.

The lower layer includes eight detection sites for LAMP amplification, a heating plate to maintain the amplification temperature (65 °C), UV-LEDs to excite the fluorescence of the amplification products, and a power supply battery. The positions of the detection sites align with the reaction pads of the VFPT, including three for detecting HIV, HBV, and HCV, one for the internal control (IC), and four for no template control (NTC).

The upper layer features a chip cover and a sealing cover to secure the detection sites, along with a filter for observing fluorescence. The amplified nucleic acids are stained with fluorescent dye and excited by LED light. The emitted fluorescence signal is captured by a mobile phone placed above the device through the filter, and the fluorescence signal is analyzed to quantify the concentration of nucleic acids using self-developed software.

### Vertical flow model

Considering the fluid flow through the filter pad as Darcy flow with a constant permeability *k*. Hence, the volumetric flux *q* follows

$$q(t)=\frac{k}{\mu }\frac{\varDelta p(t)}{h}$$

where *μ* denotes the fluid viscosity, *h* denotes the thickness of the filter pad, and $\varDelta p$ denotes the difference of fluid pressure between the two sides of the filter pad. During the vertical flow test of the cellulose papers, the pressure gradient of the fluid is mainly caused by the remaining volume of fluid *V(t)* on the upper side of the cellulose filter papers, i.e.,

$$\varDelta p=\rho g\frac{V(t)}{S}$$

where $\rho$ denotes the density of the liquid sample, *g* denotes the local gravitational acceleration, and *S* denotes the effective permeable area of the cellulose paper. By denoting the initial fluid volume in the test as *V*_*0*_, the governing equations are closed by

$$V(t)={V}_{0}-{\int }_{0}^{t}q(t){Sdt}$$

With these governing equations, the remaining volume of the liquid sample *V(t)* during the vertical flow test of the cellulose paper is determined as

$$V(t)={V}_{0}\exp \left(-\frac{\rho gk}{\mu h}t\right)$$

In the vertical flow test, once the volume of the remaining liquid is lower than a specific threshold ${V}_{{end}}=\phi {V}_{0}$, the test is stopped and the average volumetric flow rate $\bar{v}=\bar{q}S$ of the vertical flow is calculated

$$\bar{v}=-\frac{\mu {V}_{0}h}{\rho gk\,{\mathrm{ln}}\,\phi }$$

With different types and layers of the cellulose filter papers used in the test, the permeabilities of the cellulose papers are calibrated, and the average flow rates in the experiments and our model are compared.

### Horizontal flow model

In the horizontal case, considering the fluid sample diffuses in the x-axis, the horizontal volumetric fluid flux *q(x,t)* follows Darcy’s law and the principle of conservation of mass

$$q(x,t)=-\frac{k}{\mu }\frac{\partial p(x,t)}{\partial x}$$

$$\frac{\partial q(x,t)}{\partial x}+\gamma =0$$

where $\gamma$ denotes the volume of fluid required to infiltrate a unit length of cellulose paper. The remaining volume of the liquid sample *V(t)* at the dripped side is constrained by

$$V(t)={V}_{0}-{\int }_{0}^{t}q(0,t){S}_{1}{dt}$$

where *S*_*1*_ denotes the vertical cross section of the cellulose paper. Since the length of the fluid saturated paper *L(t)* varies with time, the horizontal diffusion model becomes a Stefan problem with a moving boundary of two sides: the fluid injection side *x* = *0* and the fluid tip side *x* = *L(t)*. The boundary conditions of the horizontal diffusion equation are described as following. At the fluid injection side, the remaining liquid sample *V(t)* controls the fluid pressure:

$$p(0,t)=\rho g\frac{V(t)}{{S}_{0}}$$

where *S*_*0*_ denotes the initial area occupied by the fluid drip. At the fluid tip, we assume the fluid pressure equals to the atmosphere

$$p(L(t),t)=0$$

The saturated fluid length *L(t)* follows the initial condition *L(0)* = *0* and the Stefan condition.

$$\frac{{dL}(t)}{{dt}}\gamma {S}_{1}=-\frac{{dV}(t)}{{dt}}$$

After substituting the fluid length *L(t)* into the fluid pressure on the dripped side, the governing equation of the remaining volume of the fluid is constrained by

$$-\frac{\mu }{k{S}_{1}}\frac{{dV}}{{dt}}\left(\frac{{V}_{0}-V(t)}{\gamma {S}_{1}}\right)=\rho g\frac{V(t)}{{S}_{0}}$$

The governing equation could be solved with the initial condition *V(0)* = *V*_*0*_, and the solution is

$$V(t)=-{V}_{0}{W}_{0}\left[-\exp \left(-1-\frac{\rho g\gamma k{S}_{1}^{2}}{\mu {S}_{0}{V}_{0}}t\right)\right]$$

where *W*_*0*_*(z)* denotes the Lambert W function, which is the solution to *We*^*w*^ = *z*.

The fluid-saturated length is

$$L(t)=\frac{{V}_{0}}{\gamma {S}_{1}}\left\{1+{W}_{0}\left[-\exp \left(-1-\frac{\rho g\gamma k{S}_{1}^{2}}{\mu {S}_{0}{V}_{0}}t\right)\right]\right\}$$

### Nucleic acid extraction operation by the VFPT

In combination with the PAP and smartphone analysis software, the VFPT enables accurate quantitative nucleic acid detection in the field through four steps:

For the sample incubation step, a 450 μL whole blood sample is mixed with 1500 μL lysate and dropped onto the filter pad (pink) of the folded VFPT. We incubated the system at room temperature (25 °C) for 3 minutes, to ensure the complete absorption of the sample by the filter pad and transfer of nucleic acids to the capture pad. This operation filters cell debris while captures nucleic acids onto the capture pad (orange). Waste liquids continue to flow downstream and are adsorbed by the waste liquid pad at the bottom.

For the washing buffer step, 500 μL washing buffer is added to the capture pad and incubated for 2 minutes to ensure thorough removal of impurities (e.g., proteins, cell debris) from the capture pad while avoiding excessive loss of adsorbed nucleic acids.

Then, the capture pad is folded onto the detection pads, and 50 μL DEPC-ddH₂O is added to elute the nucleic acids onto the folded multi-layer detection pads.

Finally, the folded detection pads are flattened and transferred to the portable analytical device for LAMP amplification.

In the PAP, species-specific LAMP reagents are prepackaged on the detection sites. Once the detection pads are aligned with the detection sites, the cellulose filter papers of the detection pads are pressed into the detection chamber through the chip cover and sealed with the sealing cover to prevent evaporation. The nucleic acids are amplified in the LAMP reagents at a constant temperature of 65 °C, provided by the embedded heating plate. After 60 minutes, fluorescent signals triggered by the UV LEDs are captured by the smartphone to display the detection results. The fluorescence intensity is analyzed through the self-developed software, and the quantitative results are automatically displayed.

Thus, the low-cost VFPT, the homemade PAP, and a smartphone enable point-of-care nucleic acid testing.

### Comparison of the vertical flow assay (VFA) and the lateral flow assay (LFA)

By utilizing the vertical flow assay, the VFPT facilitates longer mass transportation of liquid within the filter papers, combining gravitational and capillary forces. This allows nucleic acid molecules in the liquid to be transported to more layers of detection pads, resulting in higher detection throughput. Fluorescent molecules were used to compare the vertical and lateral flow of the liquid on the cellulose filter papers, and the transportation distances in both schemes were analyzed.

In the vertical flow scheme, multiple layers of cellulose filter papers, each with a side length of 6 mm, were arranged vertically. In the lateral flow scheme, the cellulose filter papers were placed in parallel. Then, 20 μL of fluorescent molecules, including nucleic acids, proteins, and sodium fluorescein, were added to the first layer. After allowing the liquid to fully diffuse for 5 minutes, the fluorescence intensity of each layer was measured using a multifunctional microplate detector (Tecan Spark, Männedorf, Switzerland) to analyze the liquid transportation distance and molecular transfer efficiency. Additionally, fluorescence images of each layer during the vertical flow were captured using fluorescence microscopy (Olympus IX83, Tokyo, Japan).

Comparison experiments of vertical and lateral flow were conducted on various types of filter papers, with pore sizes ranging from 1 μm to 25 μm (including 2.5 μm, 3 μm, 6 μm, 8 μm, and 11 μm).

### Comparison of actual performance between VFA and LFA

Applications based on the VFPT device and wax printing papers were used to compare the actual performance of vertical flow (VFA) and lateral flow (LFA). As the most commonly used lateral flow scheme, wax paper-based devices have been presented in many studies with different configurations, mainly divided into three types (using three detection pads as an example): Starting from the central detection pad, the liquid diffuses directly to the other two detection pads (L1). Starting from the central detection pad, the liquid diagonally diffuses to the other two detection pads (L2). Starting from a central point, the liquid diffuses to all three detection pads (L3).

For the vertical flow, the liquid is dropped directly onto the folded three-layer VFPT device, allowing for rapid downward diffusion under the influence of gravity and capillary forces (V1). Cellulose filter papers with a diameter of 6 mm and an aperture of 8 μm were used as the detection pads. The microchannels connected to the detection pads were designed to be 1.5 mm wide and 3 mm long.

20 μL of red ink was dropped onto these devices to quantify the liquid transportation distance. After the liquid had fully diffused (5 minutes), the red grayscale value of each detection pad was analyzed using ImageJ software. Additionally, 20 μL of sodium fluorescein solution was added to quantify the molecular transfer efficiency. After the liquid had fully diffused (5 minutes), the average fluorescence intensity of each detection pad was analyzed using ImageJ software.

Finally, 20 μL of HBV DNA solution was applied to these devices to assess the effect of different flow modes on the actual performance of nucleic acid amplification. After the liquid had fully diffused (5 minutes), the nucleic acids at each detection pad were eluted with DEPC-ddH2O for PCR amplification.

### The filtration performance of VFPT

VFPT has the ability to process original samples, allowing the filter pad to retain cell debris from whole blood while enabling molecules such as nucleic acids to flow to the capture pad with the liquid. Whole rabbit blood was used as the original sample to investigate the filtration function of VFPT. In a mixture of 450 μL of rabbit whole blood and HBV DNA, 2.5 μL of 1 mM DiD fluorescent dye was added to stain the cell membranes (incubated at 37 °C for 15 minutes). Then, 1500 μL of cell lysate was added, followed by 50 μL of SYBR Green-labeled nucleic acid. This mixture was then added to the VFPT for cell debris filtration and nucleic acid extraction. Fluorescence microscopy was used to observe the fluorescence on the filter pad, capture pad, and waste liquid pad, verifying that cell fragments stained with DiD were trapped by the filter pad, while nucleic acids stained with SYBR Green were adsorbed by the capture pad.

For filter pad type selection, 450 μL of whole blood samples mixed with HBV DNA were filtered using cellulose filter papers with different pore sizes (25 μm, 11 μm, 8 μm, 6 μm, 3 μm, 2.5 μm, 1 μm) and layers (1 layer, 2 layers, 3 layers). After the HBV DNA was adsorbed onto the capture pad, it was eluted with 50 μL of DEPC-ddH2O and used for subsequent PCR amplification to compare the effects of different types of cellulose filter paper on nucleic acid extraction and amplification. Additionally, the filtration time was assessed to evaluate the effect of different types of cellulose filter paper on the flow rate of the liquid.

For organisms involved in cross-experiments (including Mycobacterium tuberculosis, Ureaplasma urealyticum, Chlamydia trachomatis, Gonococcus, and Group B Streptococcus), a species-specific lysis strategy was adopted based on their cell wall/membrane characteristics to ensure efficient nucleic acid release:

For most gram-positive/negative bacteria (e.g., Group B Streptococcus, Gonococcus), the lysis buffer used for clinical plasma samples (supplied by Beiwo, Hangzhou, China) was slightly modified by adding 1 mg/mL lysozyme (to degrade peptidoglycan in the cell wall). The bacterial suspension and modified lysis buffer were mixed and incubated at 37 °C for 5 minutes to achieve complete lysis.

For Mycobacterium tuberculosis, with a thick, lipid-rich cell wall resistant to conventional lysis, an optimized protocol was applied: (i) a specialized lysis buffer containing 2% (v/v) Triton X-100 and 0.5 mg/mL lysozyme was used to disrupt lipid layers; (ii) the mixture was incubated at 55 °C for 10 minutes to enhance lipid solubilization and cell wall breakdown; (iii) 0.1 M NaOH was added and incubated at room temperature for 2 minutes to further lyse residual intact cells.

### Nucleic acid extraction performance of VFPT

In the extraction process of the VFPT, the nucleic acid in the sample flows through the filter pad with the liquid and is specifically adsorbed by the capture pad made of silicon film, completing the extraction process. The number of layers of silicon film affects the extraction performance of nucleic acid. A whole blood sample of 450 μL mixed with HBV DNA was added to the VFPT, and after the cell debris was removed by the filter pad, the DNA was adsorbed by the capture pad with varying layers (1 layer, 2 layers, 3 layers, 4 layers). The DNA was then eluted with 50 μL of DEPC-ddH2O and used for subsequent PCR amplification. The optimal number of capture pad layers was determined by comparing the CT values of the DNA eluted from different capture pad configurations.

### Comparison of different extraction methods

After optimizing the VFPT, the extraction efficiency was compared with that of commercial methods, including the column centrifugal method and the magnetic bead method. Fifty microliters of HBV DNA at different concentrations (10³, 10⁴, 10⁷ copies/mL) were mixed into 450 μL of rabbit whole blood as the original sample and extracted using the three methods.

For the VFPT method: The 450 μL sample was mixed with 1500 μL of lysate and added to the filter pad of the VFPT to remove cell debris, allowing the nucleic acid to be captured by the capture pad. After washing with 500 μL of washing buffer, 50 μL of DEPC-ddH2O was added to elute the DNA from the capture pad for subsequent PCR amplification.

### The extraction efficiency of VFPT

Different concentrations of HBV DNA were respectively used to explore the extraction efficiency of VFPT. 50 μL 10^6^ copies/mL of HBV DNA (high concentration) and 50 μL 10^3^ copies/mL of HBV DNA (low concentration) were mixed into 500 μL of rabbit whole blood, respectively, as the original sample. Then the 450 μL sample was mixed with 1500 μL lysate and added to the filter pad of the VFPT. After filtration and washing, 50 μL DEPC-ddH_2_O was added to elute the DNA on the capture pad for subsequent PCR amplification. The extraction efficiency is defined as the ratio of the CT values of the DNA after extraction and the CT values of the pure DNA without extraction.

### Optimization of detection pads

The extracted nucleic acids were eluted onto the detection pads for further amplification and analysis by adding an elution buffer to the capture pad. Utilizing the vertical flow method, the nucleic acids were transferred between detection sites on different layers, enabling scalable multiplexed detection.

Cellulose filter papers with different pore sizes (25 μm, 11 μm, 8 μm, 6 μm, 3 μm, and 2.5 μm) were found to influence both the liquid carrying capacity and the nucleic acid adsorption efficiency.

To assess the liquid carrying capacity of the cellulose filter paper, a circular filter paper with a diameter of 125 mm was used for a water absorption experiment in a Cobb instrument. The Cobb value is a standard metric to evaluate the liquid absorption capacity of cellulose filter papers for detection pads, representing the mass of water absorbed per unit area (g/m^2^) of the filter paper within a specified time. It is calculated using the formula:

$$C=({M}_{2}-{M}_{1})\times 100g/{m}^{2}$$

Where *M*_*1*_ and *M*_*2*_ are the masses of the filter paper before and after water absorption, respectively.

For the nucleic acid adsorption on each layer of the detection pad, 20 μL of fluorescent nucleic acid was added to the ten-layer filter paper forming the detection pad, and the fluorescence value of each layer of cellulose filter paper was detected by the multifunctional microplate detector (Tecan spark, Männedorf, Switzerland). In addition, 20 μL 10^3^ copies/mL of HBV DNA was added to the three-layer folded detection pads. After the liquid was fully diffused (5 minutes), each layer of the cellulose filter paper was eluted with 50 μL DEPC-ddH_2_O, and then used for PCR amplification. The nucleic acid adsorption capacity of cellulose filter paper with different pore sizes was optimized by analyzing the CT values.

### Analytical sensitivity and specificity of VFPT

Specific LAMP primers were designed and optimized according to the specific sequences of HIV, HBV, and HCV, as shown in Table S1. After the amplification of these primers, the specific bands of the amplified products were analyzed by agarose gel electrophoresis, and further confirmed by PCR sequencing (Sangon Biotech). In addition, Human papillomavirus, Influenza A virus, Influenza B virus, Ureaplasma urealyticum, Chlamydia trachomatis, Gonococcus, Mycobacterium tuberculosis, and Group B streptococcus were used to perform cross-experiments to show that the specific primers can only amplify the corresponding species.

For the sensitivity of VFPT detection of HIV, HBV, and HCV, 900 μL 10^2^-10^8^ copies/mL of plasma-based standards were mixed with lysate, added to VFPT for nucleic acid extraction, and eluted on the detection pads with 50 μL DEPC-ddH_2_O. Then, the detection pads were placed in the portable analytical device and performed LAMP amplification at the detection sites. After 60 minutes of reaction, the images of HIV, HBV, and HCV detection sites were collected, and the fluorescence intensity was analyzed by the ImageJ software, compared with the self-developed fluorescence quantitative software. GAPDH was used as a reference gene for positive control to judge the validity of nucleic acid extraction.

For the bench PCR and LAMP experiments, nucleic acid was extracted from the detection pads and eluted with 50 μL DEPC-ddH_2_O, and then 5 μL of which was used as the reaction template for subsequent amplification.

### Clinical sample detection by VFPT

We examined 200 clinical samples provided by the West China Hospital, Sichuan University, and Yibin Hospital. After mixing the obtained blood sample with the lysate, it was added directly to the filter pad of the VFPT to filter the cell debris and enable the nucleic acid to adsorb to the capture pad. Then, added 500 μL of washing buffer to remove impurities of the capture pad. Next, 50 μL of DEPC-ddH_2_O was added to remove the nucleic acid to the detection pads. Finally, the detection pads were placed on the portable analytical device for LAMP amplification. The reaction results were photographed by mobile phone and analyzed by the self-developed fluorescence quantitative software. At the same time, the laboratory PCR results were used as head-to-head analysis to determine the clinical sensitivity of the VFPT.

### Nucleic acid amplification by qPCR

Taq Pro HS Probe Master Mix was used for qPCR amplification (VazymePN101, Nanjing, China) and the specific primer sequences are shown in Table S2. Each reaction contained 4 μL 5 × Master Mix, 0.4 μL forward and reverse primers, 0.2 μL TaqMan probes, 0.2 μL Taq Pro HS DNA Polymerase, 5 μL DNA template. qPCR was performed using a Bio-rad CFX96 thermal cycler (California, USA). The thermal cycle program was preheated at 37 °C for 2 minutes, 95 °C for 30 seconds, followed by 40 cycles of 95 °C denaturation for 10 seconds, 60 °C annealing, and extension for 30 seconds.

### Nucleic acid amplification by LAMP/RT-LAMP

In the LAMP reaction system, the final concentration of each component was optimized: BST 3.0 DNA/RNA polymerase 0.32 U/μL, reverse transcriptase 2 U/μL, RNA inhibitor 2 U/μL, external primer of F3/B3 0.2 μM, internal primer of FIP/BIP 1.6 μM, ring primer of LF/LB 0.8 μM, MgSO_4_ 6 mM, KCl 50 mM, dNTPs 1.2 mM, Evagreen fluorescent dye 1X, NaOH 2 mM, Tween 20 0.1% (v/v), and template 5 μL. The reaction temperature was 65 °C and the reaction time was 60 minutes.

For the paper-based LAMP amplification reaction, the lyophilized LAMP reaction master mix was pre-deposited on the detection pads of the VFPT. The pretreatment process involved dissolving the LAMP master mix components in a trehalose-containing buffer (10% w/v trehalose, used as a cryoprotectant and stabilizer) to form a homogeneous solution. A volume of 5 μL of this solution was then spotted onto each detection pads under sterile conditions, followed by lyophilization at -40 °C for 2 hours using a freeze-dryer (Labconco FreeZone 2.5, USA) to convert the liquid master mix into a stable dry film. The dry master mix can rapidly re-hydrate within 1 minute to initiate the LAMP reaction at 65 °C, eliminating the need for on-site preparation of reaction reagents and enhancing the VFPT system’s user-friendliness for point-of-care settings.

### Statistical analyses

Statistical analyses were performed by ANOVA, P values < 0.05 were believed to be statistically significant. Data analysis was performed using Origin 9.1 and GraphPad Prism 8.0 software. The image results were analyzed using ImageJ software. For the sensitivity and specificity of the clinical specimens, sensitivity = TP/(TP + FN), specificity = TN/(TN + FP) (TP was the number of true positive samples; TN was the number of true negative samples; FP was the number of false positive samples; FN was the number of false negative samples.

## Supplementary information

supplementary materials

The production method of roll-to-roll manufacturing

The customized intelligent app quantitatively results and displays them on the screen
