# Supplementary material for: Vertical-flow tearable paper-tape rolls for scalable multiplexed point-of-care nucleic acid testing
Source: Microsyst Nanoeng. 2026 Apr 7;12:120. doi: 10.1038/s41378-026-01172-w (PMC13056913; doi:10.1038/s41378-026-01172-w)
Supplement: Supplementary file 1 — supplementary materials [file 41378_2026_1172_MOESM1_ESM.docx]

Vertical-Flow Tearable Paper-Tape Rolls for Scalable Multiplexed Point-of-Care Nucleic Acid Testing

This PDF file includes:

Figures S1 to S8

Tables S1 to S2

Movies S1 to S2


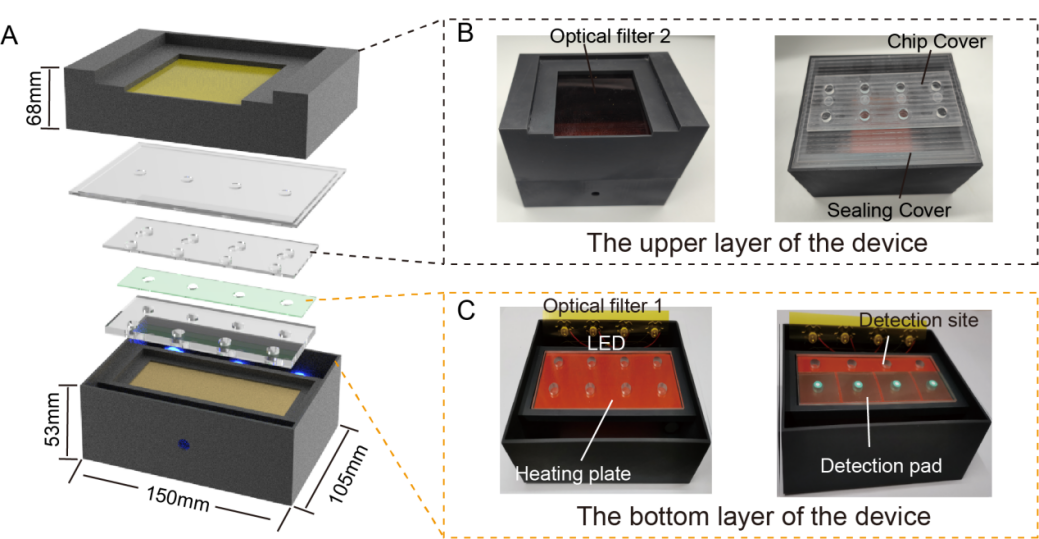


**Figure S1.** Overall structure of the VFPT analysis platform. (A) Hierarchical structure of VFPT platform, including the flattened multiplexed detection pads and the home-made portable analysis platform. The flattened multiplexed detection pad was placed on a self-made portable analysis platform for isothermal amplification. (B) The upper layer of the portable analysis platform. Includes a sealing cover and a chip cover for sealing the detection chamber, as well as optical filter 2 for mobile phone camera observation. (C) The bottom layer of the portable analysis platform. Includes a heating plate for providing temperature, a detection chip with detection sites for the LAMP reaction, and four UV-LEDs for exciting the fluorescence of the LAMP system.


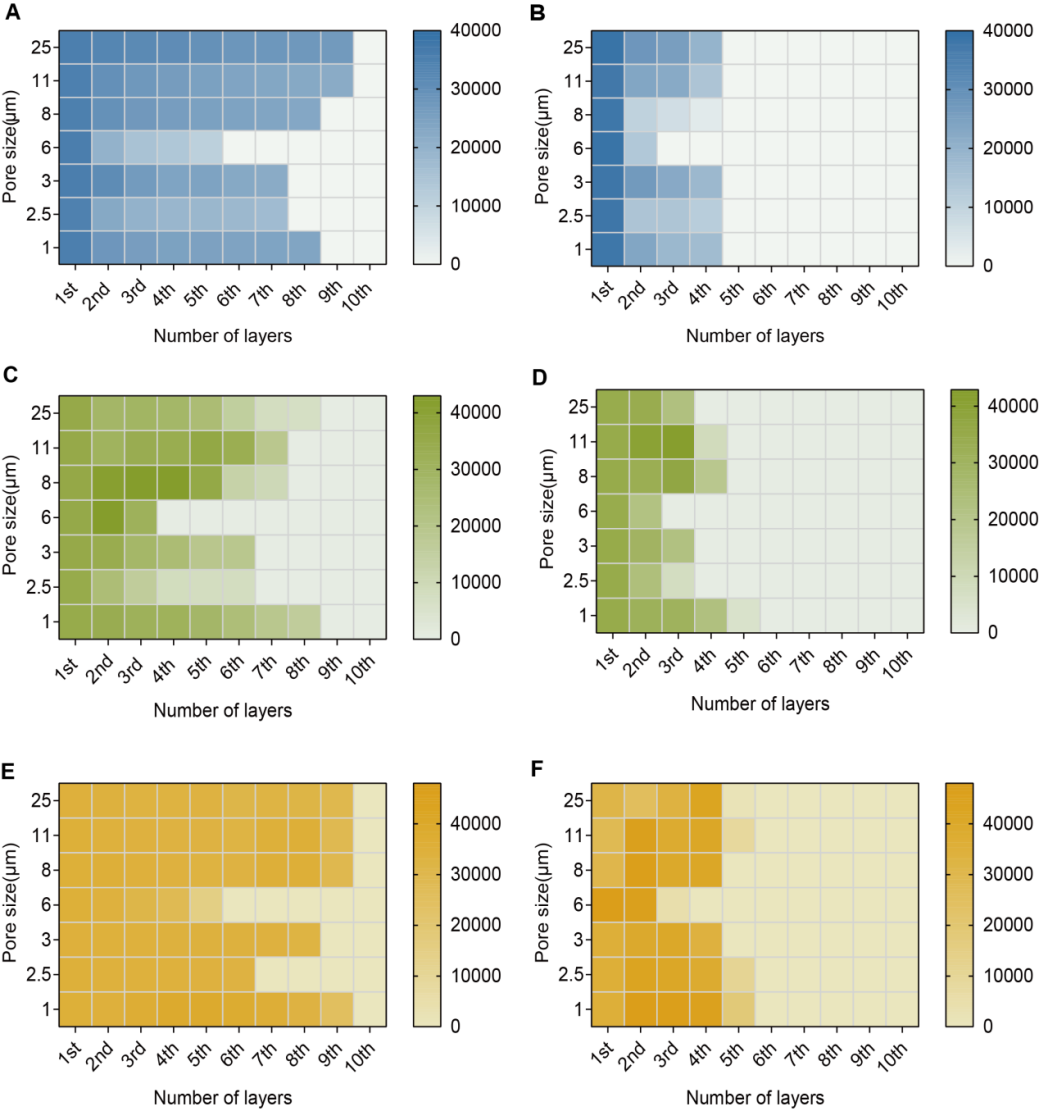


**Figure S2.** Transportation distance of the vertical way and the lateral way on different filter papers. (A) The transportation distance of fluorescently-labelled nucleic acids in the vertical way. (B) The transportation distance of nucleic acids in the lateral way. (C) The transportation distance of proteins in the vertical way. (D) The transportation distance of proteins in the lateral way. (E) The transportation distance of fluorescein sodium in the vertical way. (F) The transportation distance of fluorescein sodium in the lateral way.


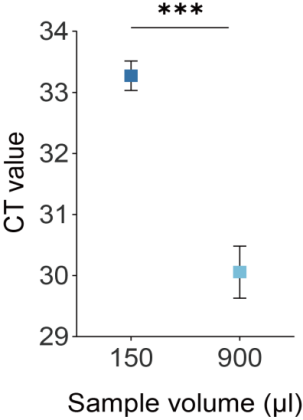


**Figure S3.** Volume of liquid sample processed by VFPT. With the sample volume increasing from 150 μl to 900 μl, which is 6 times higher, the CT values of VFPT decreases from 33.61 to 30.06.

**
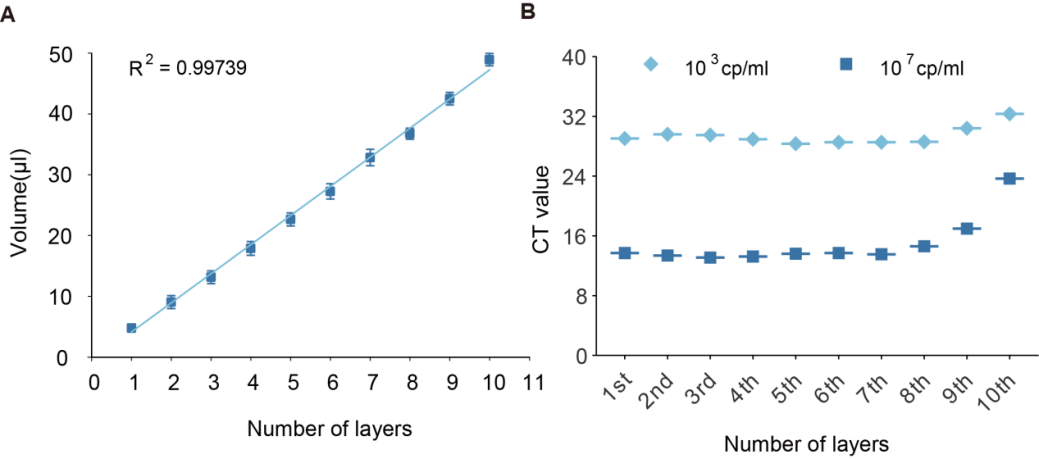
**

**Figure S4.** The optimization of the detection pads. (A) Liquid carrying capacity of different layers of the detection pads. (B) The nucleic acid adsorption capacity of each layer of the detection pads.


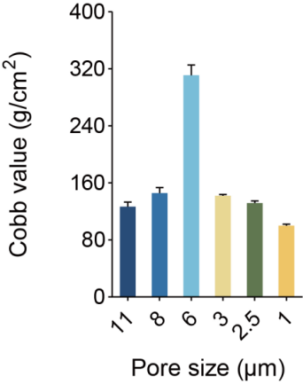


**Figure S5.** Liquid carrying capacity of filter paper with different pore sizes.Filter paper with a pore size of 6 μm has a significantly higher Cobb value than others.

**
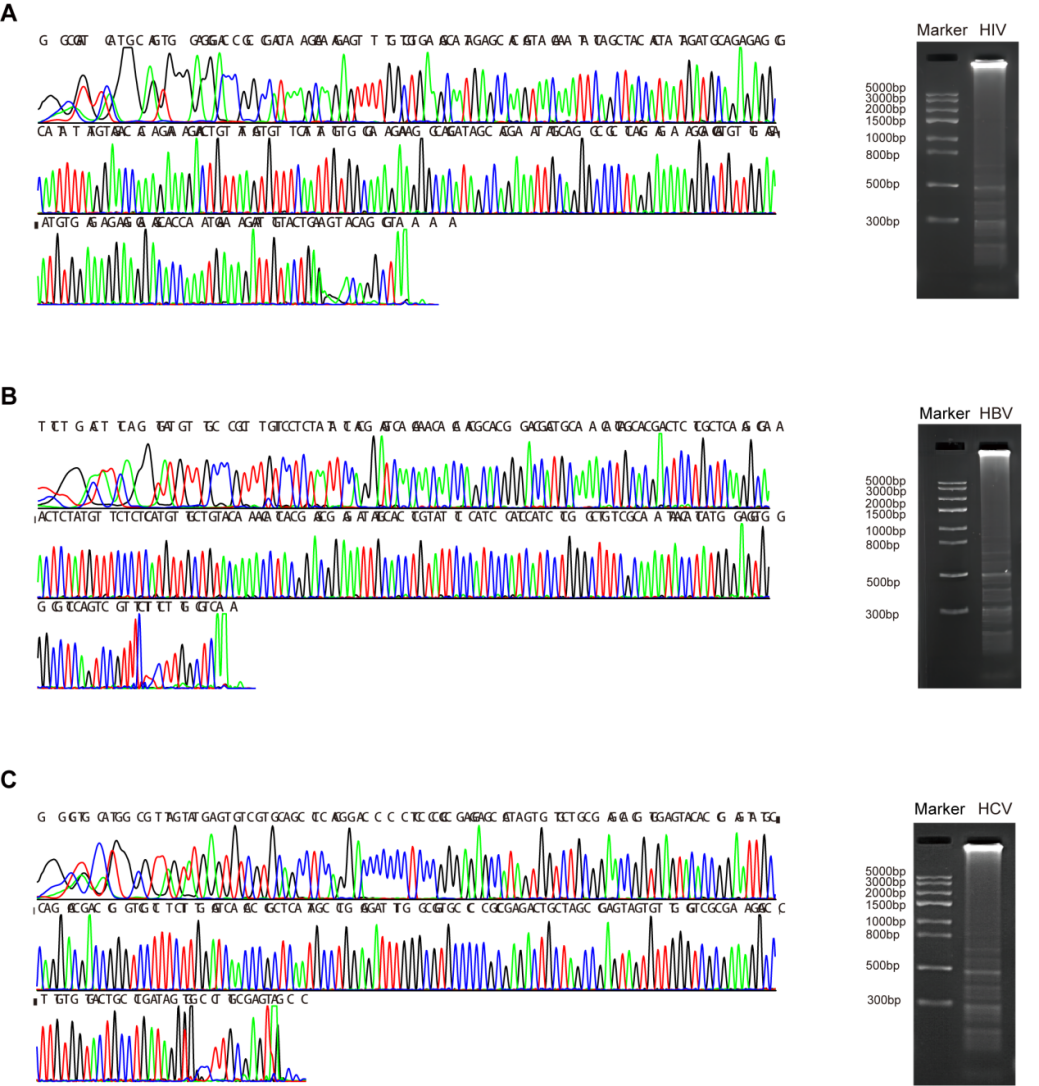
**

**Figure S6. The design and correctness verification of LAMP-specific primers**. The specific sequence and agarose gel electrophoresis result of HIV LAMP primers (A), HBV LAMP primers (B), and HCV LAMP primers. Directly confirms that the designed LAMP primers can specifically amplify the corresponding target sequences.


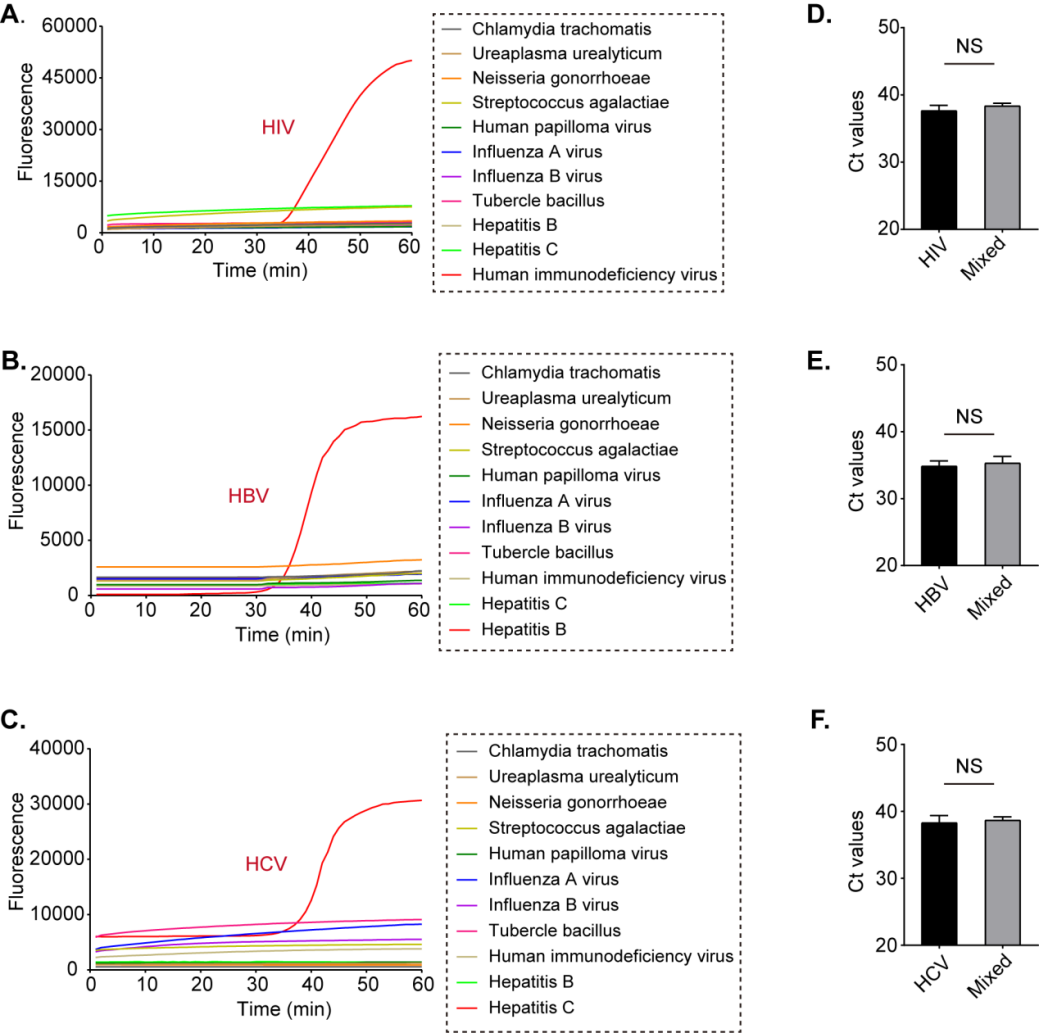


**Figure S7. Cross-reactivity detection of LAMP-specific primers**. (A) Cross-detection results of HIV LAMP primers with nucleic acids from non-target organisms. (B) Cross-detection results of HBV LAMP primers with nucleic acids from non-target organisms. (C) Cross-detection results of HCV LAMP primers with nucleic acids from non-target organisms. (D) Specific amplification of the mixed nucleic acids by HIV LAMP primers. (E) Specific amplification of the mixed nucleic acids by HBV LAMP primers. (F) Specific amplification of the mixed nucleic acids by HCV LAMP primers. All experiments were performed based on real-time LAMP amplification curves. The results show no significant amplification signals when the primers react with nucleic acids from non-relevant organisms, while specific amplification is observed for the target sequences even in the mixed nucleic acid system.


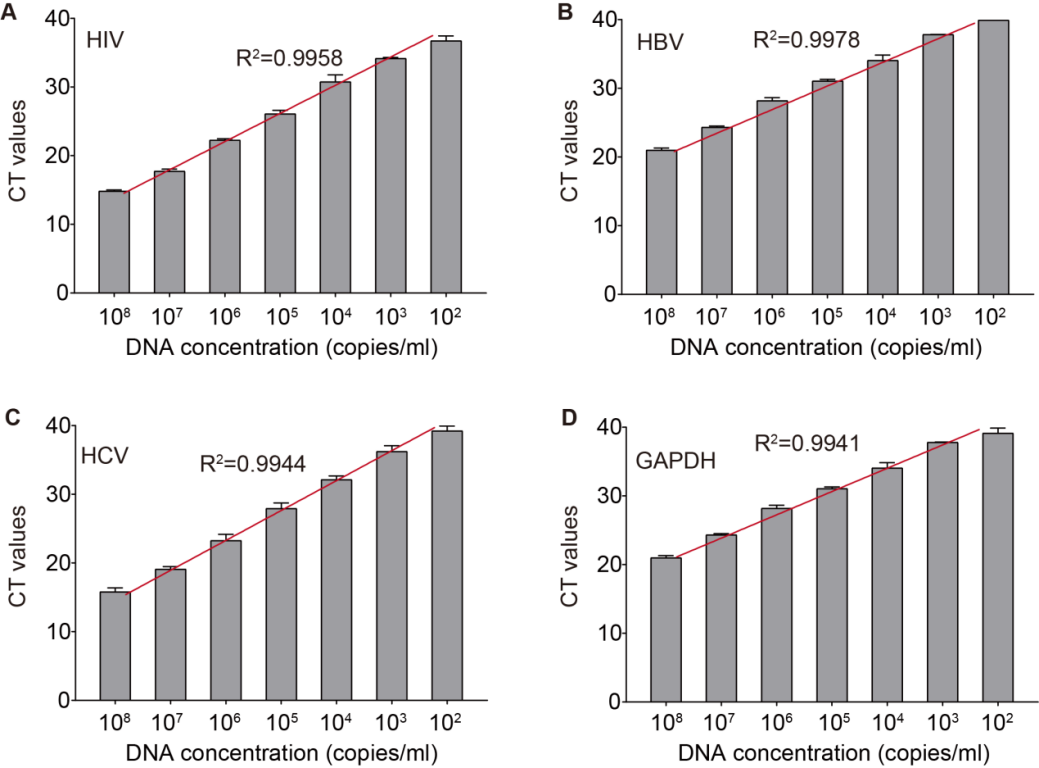


**Figure S8.** PCR amplification of each target under varying template concentrations. (A) PCR amplification of the HIV PCR primers. (B) PCR amplification of the HBV PCR primers. (C) PCR amplification of the HCV PCR primers. (D) PCR amplification of the GAPDH PCR primers. (All Ct values presented herein are derived from qPCR experiments, and are used to compare with the results of LAMP amplification for the same target sequence at different concentrations)

**Table S1.** The sequences of primers for LAMP assay.

| **Object** | **Target gene** | **Sequence** | |
| --- | --- | --- | --- |
| HIV | gag | F3 | GCAGGCTACACTAGAAGA |
|  |  | B3 | TAGCCTGTCTCTCAGTAC |
|  |  | FIP | GCCTCTCTGCATCATTATGGTAGAGTTTTGGCTGAAGCAA |
|  |  | BIP | GTTTCAATTGTGGCAAAGAAGGGAACAGCCCTTTTTCCTAG |
|  |  | LB | ACATCAGCCAGAAATTGCAGGG |
| HBV | S | F3 | TGCTGCTATGCCTCATCT |
|  |  | B3 | TGAGCCAAGAGAAACGGA |
|  |  | FIP | TGCAGGTTTTGCATGGTCCCATCAAGGTATGTTGCCCGT |
|  |  | BIP | CGACTCCTGCTCAAGGCAACAATACAGGTGCAATTTCCGT |
|  |  | LF | GCTGGTTGTTGTTGATCCTGGAAT |
|  |  | LB | CATGTTGCTGTACAAAACCTACGG |
| HCV | 5UTR | F3 | GGAACTACTGTCTTCACGC |
|  |  | B3 | ACTCGCAAGCACCCTATC |
|  |  | FIP | CGGTGTACTCACCGGTTCCGTATGAGTGTCGTGCAGCC |
|  |  | BIP | CTTGGATCAACCCGCTCAATGCCACTACTCGGCTAGCAGTC |
|  |  | LF | CAGACCACTATGGCTCTCCC |
|  |  | LB | CTGGAGATTTGGGCGTGC |

**Table S2.** The sequences of primers for PCR assay.

| **Object** | **Sequence** | |
| --- | --- | --- |
| HBV | Forward primer1 | GAATCCTCACAATACCGCAGAGT |
|  | Reverse primer1 | GCCAAGACACACGGGTGAT |
|  | Probe | 5'FAM-AAGTCCACCACGAGTCTAG-3'MGB |
|  | Forward primer1 | ATCACTCACCAACCTCCT |
|  | Reverse primer1 | TAGTCCAGAAGAACCAACAA |

**Table S3.** Comparison of different flow modes for paper-based DNA detection.

| **Flow type** | **Detection throughput** | **Mulitplexed**  **capability** | **Sensitivity** | **Cost** | **Fabrication method** |
| --- | --- | --- | --- | --- | --- |
| vertical flow  in this study | scalable | 3~10 tests | 200 copies/mL | $ 0.10 | roll-to-roll mass production |
| lateral flow ^1^ | fixed | 3 tests | 10^5^ IU/mL | $ 2.00 | wax printing |
| lateral flow ^2^ | fixed | 4 tests | 10^5^ IU/mL | $ 2.00 | wax printing |
| lateral flow ^3^ | fixed | 1 test | 500 copies/mL | $ 2.00 | commercialized |
| paper folding ^4^ | scalable | 8 tests | 400 copies/μL | $ 0.30 | wax printing |
| lateral flow ^5^ | fixed | 5 tests | 115-274 copies/μL | - | wax printing |
| lateral flow ^6^ | fixed | 1 test | 200 copies/mL | - | wax printing |
| paper folding ^7^ | fixed | 1 test | 10^10^ copies/mL | $ 0.36 | wax printing |

**Table S4.** Comparison the VFPT system with commercial platforms

| **Platforms** | **Integration** | **Automation** | **Quantitative capabilities** | **Multiplex detection capabilities** | **Cost** | **Power** |
| --- | --- | --- | --- | --- | --- | --- |
| Cepheid Xpert | Extract and amplification | √ | qPCR | 16 | $10,0000 | AC power |
| Abbott ID NOW | Extract and amplification | √ | NEAR | 24 | $2,0000 | AC power |
| QIAGEN EZ1 | Extract | √ | - | 6 | $5,0000 | AC power |
| VFPT  (this work) | Extract and amplification | manual manipulation | LAMP | Scalable (1-8) | $0.10 | No power |

**Table S5.** The permeability values for all filter papers

| **Filter Paper Pore Size**  **(μm)** | **Thickness *h***  **(μm)** | **Permeability *k***  **(×10^-14^ m^2^)** |
| --- | --- | --- |
| 1 | 180 | 1.2 |
| 2.5 | 175 | 2.8 |
| 3 | 170 | 4.5 |
| 6 | 165 | 8.9 |
| 8 | 160 | 12.3 |
| 11 | 155 | 18.7 |
| 25 | 150 | 35.2 |

**Reference**

1. Reboud J*, et al.* Paper-based microfluidics for DNA diagnostics of malaria in low resource underserved rural communities. *Proceedings of the National Academy of Sciences* **2019**, 116, 4834-4842.

2. Xu G*, et al.* Paper-Origami-Based Multiplexed Malaria Diagnostics from Whole Blood. *Angewandte Chemie International Edition* **2016**, 55, 15250-15253.

3. Wang D*, et al.* Rapid lateral flow immunoassay for the fluorescence detection of SARS-CoV-2 RNA. *Nature Biomedical Engineering* **2020**, 4, 1150-1158.

4. Zhang T*, et al.* A paper-based assay for the colorimetric detection of SARS-CoV-2 variants at single-nucleotide resolution. *Nature Biomedical Engineering* **2022**, 6, 957-967.

5. Yang Z*, et al.* Rapid Veterinary Diagnosis of Bovine Reproductive Infectious Diseases from Semen Using Paper-Origami DNA Microfluidics. *ACS Sensors* **2018**, 3, 403-409.

6. Liu F, Zhang C. A novel paper-based microfluidic enhanced chemiluminescence biosensor for facile, reliable and highly-sensitive gene detection of Listeria monocytogenes. *Sensors and Actuators B: Chemical* **2015**, 209, 399-406.

7. Li X, Scida K, Crooks RM. Detection of Hepatitis B Virus DNA with a Paper Electrochemical Sensor. *Analytical Chemistry* **2015**, 87, 9009-9015.
